# Intraspecific Variation of the Aquatic Fungus *Articulospora tetracladia*: An Ubiquitous Perspective

**DOI:** 10.1371/journal.pone.0035884

**Published:** 2012-04-27

**Authors:** Sahadevan Seena, Sofia Duarte, Cláudia Pascoal, Fernanda Cássio

**Affiliations:** Department of Biology, Centre of Molecular and Environmental Biology (CBMA), University of Minho, Braga, Portugal; University of California Riverside, United States of America

## Abstract

The worldwide-distributed aquatic fungus *Articulospora tetracladia* Ingold is a dominant sporulating species in streams of the Northwest Iberian Peninsula. To elucidate the genetic diversity of *A. tetracladia*, we analyzed isolates collected from various types of plant litter or foam in streams from North and Central Portugal and North Spain, between 2000 and 2010. Genetic diversity of these fungal populations was assessed by denaturing gradient gel electrophoresis (DGGE) fingerprints and by using ITS1-5.8S-ITS2 barcodes. Moreover, ITS1-5.8S-ITS2 barcodes of *A. tetracladia* reported in other parts of the world (Central Europe, United Kingdom, Canada, Japan and Malaysia) were retrieved from the National Center for Biotechnology (NCBI) and the National Institute of Technology and Evaluation Biological Resource Center (NBRC) to probe into genetic diversity of *A. tetracladia*. PCR-DGGE of ITS2 region of 50 Iberian fungal isolates distinguished eight operational taxonomic units (OTUs), which were similar to those obtained from neighboring trees based on ITS2 gene sequences. On the other hand, ITS1-5.8S-ITS2 barcodes of 68 fungal isolates yielded nine OTUs, but five fungal isolates were not assigned to any of these OTUs. Molecular diversity was highest for OTU-8, which included only European isolates. Two haplotypes were observed within OTU-8 and OTU-9, while only one haplotype was found within each of the remaining OTUs. Malaysia did not share haplotypes with other countries. Overall results indicate that, apart from the Malaysian genotypes, *A. tetracladia* genotypes were geographically widespread irrespective of sampling time, sites or substrates. Furthermore, PCR-DGGE appeared to be a rapid tool for assessing intraspecific diversity of aquatic hyphomycetes.

## Introduction

Although biodiversity is commonly estimated as species number, species are not the only key component of biodiversity. It is increasingly recognized that community functions and properties are shaped by genetic diversity of the individuals constituting it [1], [2]. Indeed, genetic diversity is reported to have significant effects on primary productivity, population recovery from disturbance, intraspecific competition, and fluxes of energy and nutrients (reviewed in [3]). Therefore, disentangling genetic biodiversity might contribute to elucidating the role of biodiversity in ecosystem functioning under different environmental conditions.

Aquatic hyphomycetes are a polyphyletic group of fungi that play a key role in organic matter turnover in streams by decomposing plant litter and improving plant detritus palatability for invertebrate consumption [4]. Our current knowledge on genetic diversity, geographic differentiation, dispersal and gene flow among aquatic hyphomycete populations is limited. Peláez et al. [5] were the first to investigate intraspecific diversity of aquatic hyphomycetes in foam patches along a 1 km stream section, using random amplified polymorphic DNA (RAPD) targeting the internal transcribed spacer (ITS) gene region of the nuclear rDNA. They found 7 and 5 RAPD types in *Heliscus lugdunensis* and *Articulospora tetracladia*, respectively. Moreover, distant fungal isolates shared similar RAPD patterns, suggesting the existence of a relatively uniform pool of genotypes in a stream section in Spain [5]. On the other hand, 2 out of 13 RAPD types of *Tetrachaetum elegans* were restricted to willow leaves within a stream in France [6], while 20% of the genetic variation assessed by amplified fragment length polymorphism (AFLP) within *T. elegans* from 3 leaf types in 9 streams was explained by differences between streams in France [7]. More recently, 8 polymorphic microsatellite loci were found in *Tetracladium marchalianum* isolated from 3 rivers in USA but genetic differentiation was only observed between isolates from the most distant rivers (ca. 450 Km) [8].

*Articulospora tetracladia* Ingold [9] is a dominant sporulating species on decomposing plant litter in streams of North Portugal (56–79%, Souto Stream; 6–20%, Ave River; 32–81%, Este River, [10], [11], [12], [13], [14]) and it has been reported from other European streams (e.g. Spain [5]; France [15], [16]; Ireland [17]; Hungary [18]; Italy [19], Switzerland [20]) and streams of geographically distant areas such as Asia (e.g. Pakistan [21]; Hong Kong [22]; India [23]), North America [24]; South America (e.g. Brazil [25]; Venezuela [26]) and Oceania (e.g. New Zealand [27]). In manipulative experiments with up to five aquatic hyphomycete species, *A. tetracladia* was the most active decomposer of alder or linden leaves [28], [29], and this fungus became dominant on oak leaves at high nutrient levels [30]. Recently, it was shown that intraspecific traits of *A. tetracladia* alter biodiversity effects under metal stress: diversity effects on leaf decomposition decreased in assemblages containing a Cd-sensitive functional type of this species, but were maintained in assemblages with a Cd-resistant functional type [1].

Studies on genetic diversity within aquatic hyphomycete species have been restricted to narrow geographic locations and, consequently, the ubiquity of genotypes has not been taken into account yet. Seena et al. [31], when proposing the internal transcribed spacer gene region as a barcode for identifying aquatic hyphomycete species, found a 0.3% variation within *A. tetracladia* species. In an attempt to elucidate the genetic diversity of *A. tetracladia*, we compared 68 ITS1-5.8S-ITS2 gene sequences obtained from isolates collected from various types of plant litter or foam in streams of the Iberian Peninsula, over the last 10 years, and reported from different geographic locations at the National Center for Biotechnology (NCBI) or the National Institute of Technology and Evaluation Biological Resource Center (NBRC). Considering that denaturing gradient gel electrophoresis (DGGE) is able to discriminate fungal DNA sequences with minute differences [32], we used this fingerprint technique to distinguish *A. tetracladia* genotypes among 50 isolates from the streams of the Iberian Peninsula. We expect that our study will contribute to a better comprehension of the genetic diversity and population structure of *A. tetracladia*. We speculate that research on population ecology and evolution will greatly benefit by focusing more on genetic variability within species.

**Figure 1:**
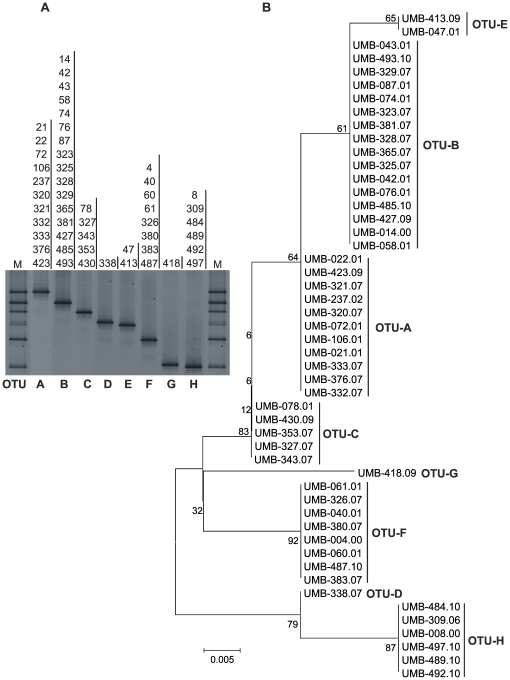
DGGE bands of the ITS2 region of rDNA produced by the 50 isolates of *A. tetracladia* (A) and neighbour joining tree based on ITS2 sequences using Kimura 2-parameter distances (B). DGGE-bands appearing at the same position on the gel or sequences grouping within the same cluster in the dendogram were considered as one OTU. The alphabets (A–H) or numbers (1–8) indicate OTUs. Bootstrap values calculated from 1000 full heuristic replicates are shown at the nodes and scale bar indicates one base change per 100 nucleotide positions of the neighbour joining tree. M, mixture of DNA of all *A. tetracladia* isolates.

**Figure 2:**
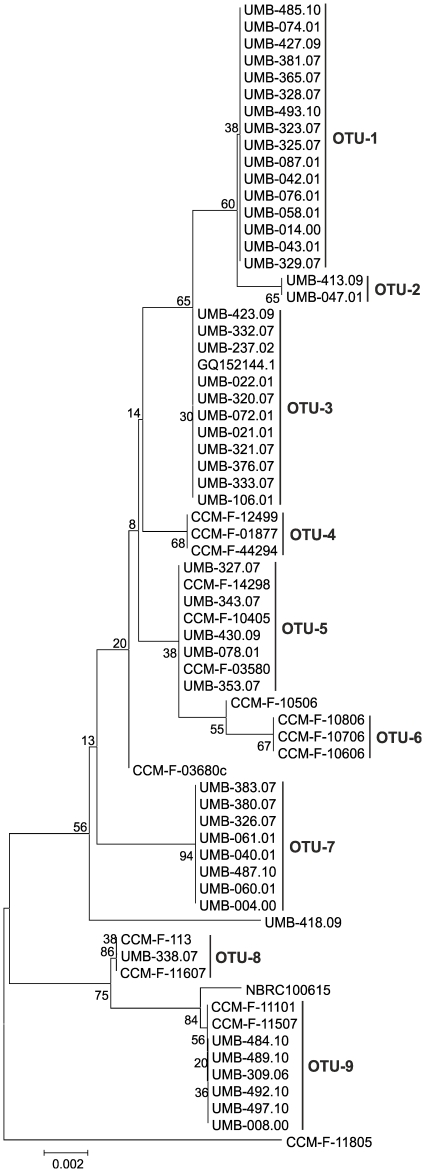
Neighbour joining tree based on ITS1-5.8S-ITS2 gene sequences using Kimura 2-parameter distances. Bootstrap values calculated from 1000 full heuristic replicates are shown at the nodes and scale bar indicates one base change per 100 nucleotide positions.

## Materials and Methods

### Fungal Isolates and Molecular Analyses

The origin of *Articulospora tetracladia* isolates, sampling date, sampled substrate (stream water, foam, leaves or twigs) and gene bank accession number of ITS1-5.8S-ITS2 sequences are listed in Table S1. Pure cultures of 50 *A. tetracladia* isolates were obtained from single spores collected in streams of the Iberian Peninsula. Fungal isolates are deposited in the culture collection of the Centre of Molecular and Environmental Biology (CBMA), Department of Biology of the University of Minho (Portugal). All cultures were grown for 2 weeks at room temperature (ca. 20°C) on 1.5% (w/v) malt extract agar. Fungal DNA was extracted with MoBio Ultraclean™ Soil DNA Isolation Kit according to manufacturer’s instruction and stored at −20°C.

ITS1-5.8S-ITS2 barcodes of the Portuguese and Spanish isolates of *A. tetracladia* that were not already in the NCBI or NBRC databases were generated as described by Seena et al. [31]. Briefly, 2 µL of DNA extract was mixed with 2 µL of ITS1 and ITS4 primers (1.6 µM final concentration of each primer) [31], 14 µL of GoTaq® Green Master Mix (Promega) and 5 µL of water supplied with the GoTaq® Green Master Mix in 0.2 mL PCR tubes. The amplification of DNA was done in a MyCycler Thermal Cycler (BioRad Laboratories, Hercules, CA, USA) using the following program: initial denaturation at 94°C for 2 min; followed by 38 cycles of denaturation at 94°C for 45 s, annealing at 55°C for 45 s and extension at 73°C for 1.5 min. Final extension was at 70°C for 10 min. The amplicons were sequenced using ITS1, ITS2 and ITS3 primers [33]. Cycle sequencing was performed using Big Dye Terminator V3.1 Kit (Applied Biosystems) according to the manufacturer’s instructions. Purified samples were denatured with formamide during 5 min at 95°C and run on an ABI 310 Genetic analyzer (Applied Biosystems) using POP4 polymer. Sequence data were deposited in GenBank (Table S1) and the alignments in TreeBASE (http://purl.org/phylo/treebase/phylows/study/TB2:S12145). Twenty-seven sequences of *A. tetracladia* from the NCBI and NBRC were also included in this study, out of which 10 sequences belong to Portuguese isolates, reported in a previous study by our group (see [31]).

For DGGE analysis, DNA from 50 Portuguese and Spanish *A. tetracladia* isolates (Table S1) was amplified with the primer pair ITS3GC/ITS4 [34], which amplifies the ITS2 region of fungal rDNA [33]. The ITS3 primer had a 40-bp GC tail at the 5′ end, which ensures separation on DGGE gels [35]. Briefly, 1 µL of fungal DNA was mixed with 0.5 µL of ITS3GC and ITS4 primers (0.4 µM final concentration of each primer) [34], 12.5 µL of GoTaq® Green Master Mix (Promega) and 10.5 µL of water supplied with the GoTaq® Green Master Mix in 0.2 mL PCR tubes. PCRs were carried out in a MyCycler Thermal Cycler using the following program: initial denaturation at 95°C of 2 min; followed by 36 cycles of denaturation at 95°C for 30 s, primer annealing at 55°C for 30 s and extension at 72°C for 1 min. Final extension was at 72°C for 5 min. The DGGE analyses were performed using a DCode™ Universal Mutation Detection System (BioRad Laboratories, Hercules, CA, USA). Five µL of each amplification product of 380–400 bp were loaded on 8% (w/v) polyacrylamide gel in 1 × TAE with a denaturing gradient from 40 to 60% (100% denaturant corresponds to 40% formamide and 7 M urea). The gels were run at 55 V, 56°C for 16 h and stained with 1 x of GelStar (Lonza) for 10 min. The gel images were captured under UV light in a gel documentation system GenoSmart (VWR, UK). A marker was prepared by mixing equal amounts of DNA of all isolates of *A. tetracladia*. DGGE-bands appearing at the same position on the gel were considered as the same operational taxonomic unit (OTU).

### Fungal DNA Barcodes and Data Analyses

Consensus sequences of ITS1-5.8S-ITS2 gene region, obtained with CodonCode Aligner Version 2.0.6 (CodonCode Co., USA), were aligned using MEGA 4 [36] and further refined with BioEdit 7.0.9 [37]. ITS1-5.8S-ITS2 sequence divergence was analysed by using Kimura 2-parameter distance [38]. The dendrogram for ITS1-5.8S-ITS2 and ITS2 gene region was generated with neighbour-joining (NJ) method using MEGA 4 [36]. All positions containing alignment gaps and missing data were eliminated only in pairwise sequence comparisons. Branch support was determined with bootstrap analysis (1000 replicates).

Bosque [39] was used for analyzing ITS1-5.8S-ITS2 to determine the operational taxonomic units (OTUs) from the similarity table (similarity % = 100). Arlequin 3.5.1.2 [40] was employed to analyse the OTUs to obtain standard indices for molecular diversity: theta S (θ_S_), theta pi (θ_π_) and pairwise F_st_ values. Theta (θ) depicts the distribution of variation within or among populations when samples are considered to represent characteristics of the larger group from which they are sampled. Theta S exhibits the infinite site equilibrium relationship between polymorphic sites, sample size and θ, for non-recombining DNA sample, while θ_π_ illustrates the infinite site equilibrium relationship between the mean number of pairwise differences and θ [41], [42]. F_st_ values based on Nei’s average number of pairwise differences [43] were calculated to measure differentiation between populations. F_st_ values range from 0 to 1, with 0 indicating no divergence between the populations, while 1 indicates that two populations are completely separated [44], [45]. Nei’s average number of pairwise differences and number of haplotypes were analysed for the OTUs and the sampling countries (Czech Republic, Portugal, Spain and Malaysia). Nei’s pairwise differences assume genetic differences that arise from mutations and changes in the frequency of an allele in a population because of random sampling.

**Figure 3:**
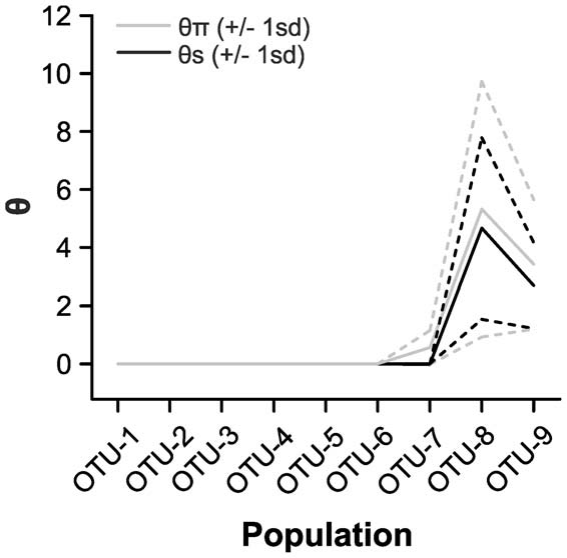
Molecular diversity indices, Theta S (θ_S_) and Theta pi (θ_π_) for all operational taxonomic units. Molecular diversity indices illustrate the level of diversity existing within the OTUs.

**Table 1:** Nucleotide composition, molecular diversity of ITS1-5.8-ITS2 sequences and haplotype frequencies within the operational taxonomic units of 68 *A. tetracladia* isolates.

Nucleotide composition (%)	OTU-1	OTU-2	OTU-3	OTU-4	OTU-5	OTU-6	OTU-7	OTU-8*	OTU-9**
C	23.28	23.28	23.49	23.28	23.71	23.49	23.90	23.29	23.53
T	29.09	29.09	29.09	29.31	29.09	29.09	28.96	29.26	28.76
A	23.71	23.49	23.71	23.49	23.28	23.49	23.04	23.51	23.53
G	23.92	24.14	23.71	23.92	23.92	23.92	24.11	23.94	24.18
**Molecular diversity**									
No. of transitions	0	0	0	0	0	0	0	2	2
No. of transversions	0	0	0	0	0	0	0	5	5
No. of substitutions	0	0	0	0	0	0	0	7	7
No. of indels	0	0	0	0	0	0	1	1	1
No. of polymorphic sites	0	0	0	0	0	0	1	8	8
No. of transition sites	0	0	0	0	0	0	0	2	2
No. of transversion sites	0	0	0	0	0	0	0	5	5
No. of substitution sites	0	0	0	0	0	0	0	7	7
No. of indel sites	0	0	0	0	0	0	1	1	1
Mean number of pairwise differences	0	0	0	0	0	0	0.57	5.33	3.43
**Haplotypes**									
Total no. of isolates	16	2	12	3	8	3	8	3	8
No. of haplotypes	1	1	1	1	1	1	1	2	2
Haplotypefrequencies	16	2	12	3	8	3	8	2, 1	6, 2

Details on fungal isolates included in each OTU are in Table S1.

* OTU-8: Haplotype 1 = CCM-F-113 and CCM-F-11607; Haplotype 2 = UMB-338.07.

** OTU-9: Haplotype 1 = UMB-008.00, UMB-309.06, UMB-484.10, UMB-489.10, UMB-492.10 and UMB-497.10; Haplotype 2 = CCM-F-11101 and CCM-F-11507.

## Results

Diversity within *Articulospora tetracladia* was assessed using rDNA gene sequences of 68 isolates (Table S1). ITS1-5.8S-ITS2 rDNA gene sequences consisted of 462–465 bp. The ITS1 region had 156–158 bp, the ITS2 region had 148–149 bp and the 5.8 S region was identical in all isolates (158 bp). Fifty one out of the 68 rDNA sequences belonged to fungal isolates collected from leaves, twigs and foam at 17 locations in 14 Iberian streams and are available in pure cultures in our collection (Table S1). Genetic diversity within these isolates of *A*. *tetracladia*, assessed by PCR-DGGE with primers targeting the ITS2 gene region of rDNA, revealed eight different OTUs (OTU-A to OTU-H, Fig. 1A). Also, the neighbour joining (NJ) tree based on ITS2 gene region yielded 8 OTUs (Fig. 1B). All isolates within each cluster of the NJ tree showed a similar migration pattern in the DGGE gel (Fig.1 A, B).

**Table 2:** Genetic distance between operational taxonomic units based on pairwise F_st_ values.

OTU	1	2	3	4	5	6	7	8	9
1	0.00								
2	1.00	0.00							
3	**1.00**	**1.00**	0.00						
4	**1.00**	1.00	**1.00**	0.00					
5	**1.00**	**1.00**	**1.00**	**1.00**	0.00				
6	**1.00**	1.00	**1.00**	**1.00**	**1.00**	0.00			
7	**0.97**	**0.91**	**0.95**	**0.90**	**0.94**	**0.93**	0.00		
8	**0.93**	0.64	**0.89**	0.60	**0.85**	0.75	**0.81**	0.00	
9	**0.92**	**0.81**	**0.90**	**0.79**	**0.88**	**0.84**	**0.86**	**0.39**	0.00

Pairwise F_st_ values of populations were tested by comparison with 95% confidence intervals from 110 permutations. Significant differences are in bold (P≤0.05).

**Table 3:** Haplotype number and frequencies between the populations of different countries.

Populations		Malaysia	CzechRepublic	Portugal	Spain
No. of isolates		4	4	4	4
No. of haplotypes		2	3	4	3
Haplotype 1	CCM-F-10706 (Malaysia)	3 (0.75)	0	0	0
	CCM-F-10606 (Malaysia)				
	CCM-F-10806 (Malaysia)				
Haplotype 2	CCM-F-10506 (Malaysia)	1 (0.25)	0	0	0
Haplotype 3	CCM-F-14298 (Czech Republic)	0	1 (0.25)	0	0
Haplotype 4	CCM-F-113 (Czech Republic)	0	1 (0.25)	1 (0.25)	0
	CCM-F-11607 (Portugal)				
Haplotype 5	CCM-F-01877 (Czech Republic)	0	2 (0.5)	0	0
	CCM-F-12499 (Czech Republic)				
Haplotype 6	UMB-329.07 (Portugal)	0	0	1 (0.25)	2 (0.5)
	UMB-485.10 (Spain)				
	UMB-493.10 (Spain)				
Haplotype 7	UMB-376.07 (Portugal)	0	0	1 (0.25)	0
Haplotype 8	UMB-047.01 (Portugal)	0	0	1 (0.25)	0
Haplotype 9	UMB-487.10 (Spain)	0	0	0	1 (0.25)
Haplotype 10	UMB-489.10 (Spain)	0	0	0	1 (0.25)

Numbers outside parenthesis denote haplotype frequency and inside represent relative frequency. Only countries with a minimum of four isolates were considered in this analysis.

**Figure 4:**
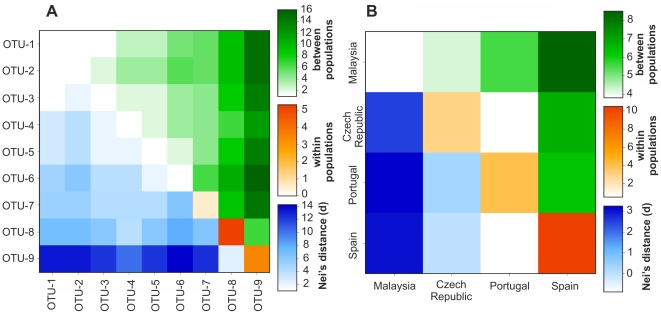
Nei’s average number of pairwise differences in OTUs (A) and populations of different countries (B). The above diagonal elements (green) denotes the Nei’s average number of pairwise differences between OTUs or sampling countries, the diagonal elements (orange) denotes Nei’s average number of pairwise differences within OTUs or sampling countries, and below diagonal elements (blue) denotes net number of nucleotide differences between OTUs or sampling countries (Nei’s distance).

The dendrogram based on ITS1-5.8S-ITS2 rDNA gene sequences of the 68 isolates of *A. tetracladia* showed distinct OTUs (Fig. 2; Table S1), supporting considerable population diversity within this species. The ITS1-5.8S-ITS2 gene sequences yielded 9 OTUs (OTU-1 to OTU-9, Table S1); five isolates were not assigned to any of the referred OTUs and included isolates from Europe, Malaysia and Japan. *Articulospora tetracladia* isolates did not exhibit cohesiveness based on sampling date or substrate (Table S1 and Fig. 2). Each OTU consisted of isolates from different geographic locations except for the OTU-6, which only included Malaysian isolates. The minimum and maximum number of isolates clustered within OTU-2 (2 isolates) and OTU-1 (16 isolates), and included Iberian and Portuguese isolates, respectively. The overall mean evolutionary divergence of sequences based on Kimura-2 parameter distance was 0.9%, the minimum and maximum divergence between the OTUs and ungrouped taxa were 0.2% and 2.2%, respectively. The evolutionary divergence between the OTUs was ≤1.3%. The evolutionary divergence between *A. tetracladia* isolates and other *Articulospora* genus, whose sequences were available in NCBI, was higher than 20% (23.3% for *A. proliferata*, accession number FJ000395.1 and 25.5% for *A. atra*, accession number FJ000402.1).

Composition of the nucleotides C, A and G in the OTU-1 to OTU-9 ranged from 23.28% to 24.18%, whereas the nucleotide T ranged from 28.76% to 29.31%. The maximum difference in nucleotide composition between the OTUs was 0.67% for A, 0.62% for C, 0.55% for T and 0.47% for G (Table 1). Molecular diversity was highest for OTU-8 followed by OTU-9 (Table 1 and Fig. 3). Theta pi (θ_π_) as the mean number of pairwise nucleotide differences was higher than theta S (θ_s_) based upon the number of polymorphic sites; however, the nucleotide diversity patterns were similar. Two haplotypes were observed within OTU-8 and OTU-9, while only one haplotype was found within each of the remaining OTUs (Table 1).

Genetic distances between OTUs provided by the F_st_ values were maxima between OTU pairs from OTU-1 to OTU-6 (F_st_ = 1.0) and lowest between OTU-8 and OTU-9 (F_st_ = 0.39, Table 2). Significant differences were found for almost all pairwise comparisons excluding OTU-2 *versus* OTU-1, OTU-4, OTU-6 or OTU-8, and OTU-8 *versus* OTU-4 or OTU-6 (Table 2).

Haplotype number and frequencies between the populations of different countries are given in Table 3. Malaysia did not share haplotypes with other countries. Czech Republic and Portugal shared the haplotypes CCM-F-113 and CCM-F-11607, while Portugal and Spain shared the haplotypes UMB-329.07, UMB-485.10 and UMB-493.10 (Table 3).

Average number of Nei’s pairwise differences obtained between and within OTUs is outlined in Fig. 4A. The maximum pairwise difference within populations was in OTU-8 (5.3) and OTU-9 (3.4), and OTU-9 exhibited the maximum pairwise difference from all other OTUs (12–16). Moreover, Nei’s average number of pairwise differences in the ITS1-5.8S-ITS2 barcodes were observed within Spanish populations and between populations in Spain and Malaysia (Fig. 4B).

## Discussion

Genetic fingerprinting techniques provide information on genetic diversity of microbial communities [35], [46]. Among them, PCR-DGGE has been widely used to assess the structure of aquatic fungal communities on decomposing plant litter in freshwaters [10], [11], [47], [48]. By using DGGE, almost 100% of the sequence variants can be detected in DNA fragments up to 500 bp [32]. In a previous study, DGGE discriminated most isolates of the gram-negative bacterium *Aeromonas* by using specific primers [49]. However, no attempts were done to explore the potential of this technique to assess intraspecific genetic diversity of aquatic fungi. In our study, PCR-DGGE of the ITS2 region of the aquatic hyphomycete *Articulospora tetracladia* led to 8 OTUs, suggesting considerable genetic diversity within this species (Fig. 1). However, only small DNA fragments (up to 500 bp) can be separated by DGGE and different DNA sequences may have similar motilities due to identical GC contents [50], which may limit our ability to assess genetic diversity.

Recently, barcodes based on the ITS/5.8 s gene region showed a high taxonomic cohesiveness for 19 aquatic hyphomycete species commonly found on decomposing plant litter in streams [31]. However, neighbour-joining (NJ) trees based only on ITS1 or ITS2 gene sequences had lower statistical support for some internal nodes and, hence, the use of ITS1-5.8S-ITS2 rRNA gene sequences was proposed for unequivocal identification of aquatic hyphomycete species. In the current study, the dendogram of ITS1-5.8S-ITS2 barcodes of 68 isolates of *A. tetracladia* from different geographic locations (e.g., Iberian Peninsula, Central Europe and East Malaysia) generated 9 clusters, which were identified as OTUs (Fig. 2). Although the NJ tree based on ITS1-5.8S-ITS2 barcodes failed to demonstrate a strong statistical support for some nodes, this observation suggests that isolates of *A. tetracladia* did not yield a single genotype but very close nonidentical populations. In our study, the maximum intraspecific evolutionary divergence between *A. tetracladia* isolates from various regions, streams or substrates was 2.2%, which agrees with values reported for fungal intraspecific variability derived from the ITS sequences (0–3%, [51]). Therefore, ITS barcodes appear to be useful to catalog the intraspecific diversity with potential application in natural environments. The NJ trees based on ITS1, ITS2 and ITS1-5.8S-ITS2 rRNA gene sequences of aquatic hyphomycete species showed similar taxonomic cohesiveness [31]. Consistently, in our study the clustering patterns of *A. tetracladia* isolates from ITS1-5.8S-ITS2 or ITS2 barcodes were identical and matched with the DGGE OTUs.

In the current study, the geographic origin of *A. tetracladia* isolates did not appear to be relevant because most isolates from the same geographic region failed to cluster together. However, OTU-6 consisted only of isolates from Malaysia (Fig. 2). The molecular diversity as shown by theta S (θ_S_) and theta pi (θ_π_) was highest for the OTU-8 (Fig. 3), which included only European isolates. These indices of molecular diversity proved to be reliable estimators of population genetic structure [45], [52]. Genetic variation within OTUs suggested that *A. tetracladia* isolates within the clusters OTU-1 to OTU-6 had no population divergence and might be generated by asexual reproduction, while those in the OTU-7 to OTU-9 are in the process of genetic differentiation (Fig. 3, Table 1). Genetic differentiation of *Tetracladium marchalianum* populations was noted between rivers separated by ca. 450 km, however evidence of sexual reproduction was not reported [8]. The F_st_ values measure differentiation between populations [44], [45], [53]. In the current study, F_st_ values lower than one were found for OTU-7 to OTU-9, suggesting that these populations may be still in the process of differentiation from the other populations (Table 2).

The maximum pairwise difference and nucleotide diversity within the OTU-7 to OTU-9 might be the result of sexual reproduction or mutation. Meiospores are considered to be responsible for long-term permanence and distance dispersal of aquatic hyphomycetes as they are sturdier and smaller than conidia [54]. Because we found that some haplotypes are shared among European streams, but not with Asian streams, we could hypothesize that genetic variation in Malaysian isolates might have resulted from long distance dispersal of a meiospore. In addition since Malaysia is an island, genetic divergence is probably more likely to occur. The role played by geographic locations on a worldwide scale, not only at the species level but also at intraspecific level needs to be further explored with multilocus phylogenetic and phylogeographic studies. Taylor et al. [55] concluded that microfungal species demonstrate biogeography and probably most of the species do not occur “everywhere” like other eukaryotic microbial species. Indeed, recent mycological studies [56], [57], [58], [59], [60] documented that fungi, like plants and animals, also have discrete distribution patterns and population structures that can be assessed and tested within a phylogenetic framework [55].

Overall, we were able to differentiate populations of *A. tetracladia* by using both DGGE fingerprinting of ITS2 gene region and comparing sequences of ITS1-5.8S-ITS2 gene region. The DGGE of the ITS2 gene region proved to be a rapid and less expensive way for assessing intraspecific diversity within the *A. tetracladia* isolates, with results obtained within a span of 5 to 16 hours. This distinguishes DGGE as a very promising tool to assess intraspecific diversity within microbial communities in aquatic ecosystems. It is acknowledged that uncertainties with regard to intraspecific variability assessment increase with the decrease in the number of conspecific ITS sequences of a species [51]. So, it also becomes critical to maintain a good culture collection of conspecific strains. The lack of multilocus gene sequences from different geographic regions in gene databases and the lack of isolates available in culture collections also limited our analysis. Further studies documenting the distribution in parallel with phylogenetic structure should be conducted in order to understand the processes shaping geographic distribution of lineages of aquatic hyphomycetes. Our study also raises an important question, as to why a significant amount of genetic diversity exists in aquatic hyphomycetes. Indeed, genetically more diverse strains of the freshwater fungi *Cylindrocarpon destructans* and *Heliscus lugdunensis*, assessed through RAPD and rDNA-ITS sequencing, produced a greater diversity of unique metabolites [61]. Does this mean that genetically diverse communities have high functional diversity or will ensure stability in the face of environmental variability? Or is it related to resource availability or to a matter of chance through history?

## Supporting Information

Table S1 Articulospora tetracladia isolate reference, stream location, date of isolation, sampled substrate and GenBank accession number within the OTUs of the 68 sequenced isolates.
(DOCX)
