# Supplementary material for: Intraspecific Variation of the Aquatic Fungus Articulospora tetracladia: An Ubiquitous Perspective
Source: PLoS One. 2012 Apr 27;7(4):e35884. doi: 10.1371/journal.pone.0035884 (PMC3338785; doi:10.1371/journal.pone.0035884)
Supplement: Table S1 — Articulospora tetracladia isolate reference, stream location, date of isolation, sampled substrate and GenBank accession number within the OTUs of the 68 sequenced isolates. (DOCX) [file pone.0035884.s001.docx]

# Supplementary Table

# Table S1. *Articulospora tetracladia* isolate reference, stream location, date of isolation, sampled substrate and GenBank accession number within the OTUs of the 68 sequenced isolates.

| OTU | Isolate reference | Year | Stream location | Country | Sampled  substrate | NCBI accession number |
| --- | --- | --- | --- | --- | --- | --- |
| OTU-1 | | | | | | |
|  | UMB-014.00 | 2000 | L6 (41°21’N 8°25’W) | Portugal | water | GQ411288^a,c^ |
|  | UMB-042.01 | 2001 | Sj (41°45’N 8°11’W) | Portugal | foam | JF895397^c^ |
|  | UMB-043.01 | 2001 | Sj (41°45’N 8°11’W) | Portugal | foam | JF895398^c^ |
|  | UMB-058.01 | 2001 | E5 (41°30’N 8°27’W) | Portugal | leaves | JF895399^c^ |
|  | UMB-074.01 | 2001 | Ma (41°46’N 8º08’W) | Portugal | foam | JF895400^c^ |
|  | UMB-076.01 | 2001 | Ma (41°46’N 8º08’W) | Portugal | foam | JF895401^c^ |
|  | UMB-087.01 | 2001 | Ma (41°46'N 8º08'W) | Portugal | foam | GQ411289^a,c^ |
|  | UMB-323.07 | 2007 | Pa (40º59’N 8º00’W) | Portugal | leaves | GQ411286^a,c^ |
|  | UMB-325.07 | 2007 | Pa (40º59’N 8º00’W) | Portugal | leaves | JF895402^c^ |
|  | UMB-328.07 | 2007 | Pa (40º59’N 8º00’W) | Portugal | leaves | GQ411287^a,c^ |
|  | UMB-329.07 | 2007 | Pa (40º59’N 8º00’W) | Portugal | leaves | JF895403^c^ |
|  | UMB-365.06 | 2006 | Ti (41°26’N 7°32’W) | Portugal | leaves | JF895404^c^ |
|  | UMB-381.06 | 2006 | Ti (41°26’N 7°32’W) | Portugal | leaves | JF895405^c^ |
|  | UMB-427.09 | 2009 | Cg (41º78’N 8º13’W | Portugal | foam | JF895406^c^ |
|  | UMB-485.10 | 2010 | Vi (42º15N 8º71’W) | Spain | foam | JF895407^c^ |
|  | UMB-493.10 | 2010 | Vi (42º15N 8º71’W) | Spain | foam | JF895408^c^ |
| OTU-2 |  |  |  |  |  |  |
|  | UMB-047.01 | 2001 | Sj (41°45’N 8°11’W) | Portugal | foam | JF895409^c^ |
|  | UMB-413.09 | 2009 | V1 (41º98’N 8º33’W) | Portugal | foam | JF895410^c^ |
| OTU-3 |  |  |  |  |  |  |
|  | UMB-021.01 | 2001 | E1 (41°34’N 8°19’W) | Portugal | foam | JF895411^c^ |
|  | UMB-022.01 | 2001 | E1 (41°34’N 8°19’W) | Portugal | foam | JF895412^c^ |
|  | UMB-072.01 | 2001 | Ma (41°46’N 8º08’W) | Portugal | foam | JF895413^c^ |
|  | UMB-106.01 | 2001 | Ma (41°46’N 8º08’W) | Portugal | foam | JF895414^c^ |
|  | UMB-237.02 | 2002 | Sj (41°45’N 8°11’W) | Portugal | foam | JF895415^c^ |
|  | UMB-320.07 | 2007 | Al (40º30’N 7º52’W) | Portugal | leaves | GQ411292^a,c^ |
|  | UMB-321.07 | 2007 | Al (40º30’N 7º52’W) | Portugal | leaves | JF895416^c^ |
|  | UMB-332.07 | 2007 | Al (40º30’N 7º52’W) | Portugal | leaves | GQ411291^a,c^ |
|  | UMB-333.07 | 2007 | Al (40º30’N 7º52’W) | Portugal | leaves | GQ411290^a,c^ |
|  | UMB-376.07 | 2006 | Pi (41°26’N 7°33’W) | Portugal | leaves | GQ411285^a,c^ |
|  | UMB-423.09 | 2009 | V2 (41º99’N 8º29’W) | Portugal | foam | JF895417^c^ |
|  | n.a. | n.a. | n.a. | Portugal | foam | GQ152144.1^a^ |
| OTU-4 |  |  |  |  |  |  |
|  | CCM-F-01877 | n.a. | n.a. | Czech Republic | foam | EU998917^a^ |
|  | CCM-F-12499 | n.a. | n.a. | Czech Republic | foam | EU998915^a^ |
|  | CCM-F-44294 | n.a. | n.a. | Canada | foam | EU998916^a^ |
| OTU-5 |  |  |  |  |  |  |
|  | UMB-078.01 | 2001 | Pr (41º45’N 8º09’W) | Portugal | dark twigs | JF895418^c^ |
|  | UMB-327.07 | 2007 | Pa (40º59’N 8º00’W) | Portugal | leaves | JF895419^c^ |
|  | UMB-343.07 | 2007 | Pa (40º59’N 8º00’W) | Portugal | leaves | GQ411293^a,c^ |
|  | UMB-353.07 | 2007 | Pa (40º59’N 8º00’W) | Portugal | leaves | JF895420^c^ |
|  | UMB-430.09 | 2009 | Ag (41º77’N 8º14’W) | Portugal | foam | JF895421^c^ |
|  | CCM-F-03580 | n.a. | n.a. | Slovak Republic | foam | EU998922^a^ |
|  | CCM F-10405 | n.a. | n.a. | Portugal | leaves | FJ000393^a^ |
|  | CCM-F-14298 | n.a. | n.a. | Czech Republic | foam | EU998923^a^ |
| OTU-6 |  |  |  |  |  |  |
|  | CCM-F-10606 | n.a. | n.a. | Malaysia | plant debris | EU998925^a^ |
|  | CCM-F-10706 | n.a. | n.a. | Malaysia | plant debris | EU998927^a^ |
|  | CCM-F-10806 | n.a. | n.a. | Malaysia | plant debris | EU998914^a^ |
| OTU-7 |  |  |  |  |  |  |
|  | UMB-004.00 | 2000 | L1 (41°33’N 8°14’W) | Portugal | water | JF895422^c^ |
|  | UMB-040.01 | 2001 | E5 (41°30’N 8°27’W) | Portugal | leaves | JF895423^c^ |
|  | UMB-060.01 | 2001 | E5 (41°30’N 8°27’W) | Portugal | leaves | JF895424^c^ |
|  | UMB-061.01 | 2001 | E5 (41°30’N 8°27’W) | Portugal | leaves | JF895425^c^ |
|  | UMB-326.07 | 2007 | Pa (40º59’N 8º00’W) | Portugal | leaves | JF895426^c^ |
|  | UMB-380.06 | 2006 | Ti (41°26’N 7°32’W) | Portugal | leaves | JF895427^c^ |
|  | UMB-383.06 | 2006 | Ti (41°26’N 7°32’W) | Portugal | leaves | JF895428^c^ |
|  | UMB-487.10 | 2010 | Vi (42º15’N 8º71’W) | Spain | foam | JF895429^c^ |
| OTU-8 |  |  |  |  |  |  |
|  | UMB-338.07 | 2007 | Pa (40º59’N 8º00’W) | Portugal | leaves | JF895432^c^ |
|  | CCM-F-113 | n.a. | n.a. | Czech Republic | foam | EU998921^a^ |
|  | CCM-F-11607 | n.a. | n.a. | Portugal (Madeira) | plant debris | EU998920^a^ |
| OTU-9 |  |  |  |  |  |  |
|  | UMB-008.00 | 2000 | L1 (41°33’N 8°14’W) | Portugal | water | JF895430^c^ |
|  | UMB-309.06 | 2006 | Ca (41°38’N 8°19’W) | Portugal | leaves | JF895431^c^ |
|  | UMB-484.10 | 2010 | Ca (41°38’N 8°19’W) | Portugal | leaves | JF895433^c^ |
|  | UMB-489.10 | 2010 | Vi (42º15’N 8º71’W) | Spain | foam | JF895434^c^ |
|  | UMB-492.10 | 2010 | Vi (42º15’N 8º71’W) | Spain | foam | JF895435^c^ |
|  | UMB-497.10 | 2010 | Vi (42º15’N 8º71’W) | Spain | foam | JF895436^c^ |
|  | CCM-F-11101 | n.a. | n.a. | United Kingdom | foam | EU998929^a^ |
|  | CCM-F-11507 | n.a. | n.a. | Portugal (Madeira) | plant debris | EU998919^a^ |
| Ungrouped |  |  |  |  |  |  |
|  | UMB-418.09 | 2009 | V2 (41º99’N 8º29’W) | Portugal | foam | JF895437^c^ |
|  | CCM-F-03680c | n.a. | n.a. | Slovak Republic | foam | EU998926^a^ |
|  | CCM-F-10506 | n.a. | n.a. | Malaysia | plant debris | EU998924^a^ |
|  | CCM-F-11805 | n.a. | n.a. | Portugal | leaves | EU998921^a^ |
|  | NBRC100615 | n.a. | n.a. | Japan | foam | NBRC100615^b^ |

Sampling sites are: Alhões Stream (Al), Ave River (L1, L6), Água da Adega Stream (Ag), Cagademos Stream (Cg), Cávado River (Ca), Este River (E1, E5), Maceira River (Ma), Patanha River (Pa), Peliteira Stream (Pi), Preguiça Stream (Pr), São João do Campo Stream (Sj), tributaries of the Vez River (V1, V2), Vigo Stream (Vi) and Tinhela Stream (Ti).

^a^, Sequences retrieved from NCBI; ^b^, Sequence retrieved from NBRC; ^c^, isolates used to perform DGGE.

n.a., not available.
